# *GLYAT* regulates JNK-mediated cell death in *Drosophila*

**DOI:** 10.1038/s41598-017-05482-y

**Published:** 2017-07-12

**Authors:** Pu Ren, Wenzhe Li, Lei Xue

**Affiliations:** Institute of Intervention Vessel, Shanghai 10th People’s Hospital, Shanghai Key Laboratory of Signaling and Diseases Research, School of Life Science and Technology, Tongji University, 1239 Siping Road, Shanghai, 200092 China

## Abstract

Cell death is a fundamental progress that regulates cell number, tissue homeostasis and organ size in development. The c-Jun N-terminal kinase (JNK) pathway has been evolutionarily conserved from fly to human, and plays essential roles in regulating cell death. To characterize additional genes that regulate JNK signaling, we performed a genetic screen in *Drosophila* and identified *dGLYAT*, a novel gene whose function was previously unknown, as a modulator of JNK-mediated cell death. We found that loss of *dGLYAT* suppressed JNK activation and cell death triggered by over-expression of Egr or Hep, or depletion of *puc* or *lgl* in development, suggesting *dGLYAT* regulates both ectopic and physiological functions of JNK pathway. Furthermore, we showed that loss of *dGLYAT* inhibits JNK-mediated ROS production, suggesting *dGLYAT* regulates multiple functions of JNK signaling *in vivo*.

## Introduction

The glycine N-acyltransferase (*GLYAT*) family, which includes *hGLYAT*, *hGLYATL1*, *hGLYATL2* and *hGLYATL3* in human^1–6^, encodes a characteristic GNAT (Gcn5-related N-Acetyltransferase) domain that is evolutionarily conserved from invertebrate to mammals^7, 8^. GLYAT proteins are specifically localized in the mitochondria^9^, and play pivotal roles in catalyzing the formation of Primary Fatty Acid Amides (PFAMs)^6, 10^, a family of bioactive lipids essential for many biological processes^6, 10, 11^. Anandamide, a member of PFAMs, was shown to activate JNK signaling and promote reactive oxygen species (ROS) formation^12–14^, yet a direct role of GLYAT in JNK signaling and cell death has not been reported. *CG34010*, whose function has not been previously investigated, encodes a *Drosophila* homolog of GLYAT, and is referred to as *dGLYAT* hereafter.

The c-Jun N-terminal kinase (JNK) signaling pathway is highly conserved from fly to mammals^15, 16^, and plays essential roles in regulating cellular activities including cell proliferation, differentiation, migration and apoptosis^17, 18^. In *Drosophila*, ectopic expression of the tumor necrosis factor (TNF) ortholog Eiger (Egr) triggers cell death through the JNK pathway^19^. Egr is recognized by its receptor Grindelwald (Grnd), which acts through the TNF receptor-associated factor2 (dTRAF2) and the Bendless (Ben)/dUev1a ubiquitin conjugating enzyme complex to initiate a kinase cascade reaction including the JNK kinase kinase dTAK1 (MAP3K), the JNK kinase hemipterous (Hep) and Basket (Bsk), the fly JNK, through phosphorylation^19–24^. The activation of JNK signaling could be reflected by the expression of its target gene *puckered* (*puc*), which encodes a JNK phosphatase that negatively regulates JNK activity and thus establishes a negative feedback loop^25–27^. In addition, activated JNK signaling promotes the production of Reactive Oxygen Species (ROS)^28, 29^.

To identify additional factors that regulate JNK-mediated cell death, we have been performing genetic screens in *Drosophila* for modifiers of Egr-triggered JNK-dependent cell death, and have characterized Ben, dUev1a and Wallenda (Wnd) as components of this evolutionary conserved pathway^24, 30, 31^. In this study, we characterized *dGLYAT* as an essential regulator of JNK signaling in *Drosophila*. Firstly, loss of *dGLYAT* suppresses ectopic Egr or Hep-induced JNK-dependent cell death in development. Secondly, depletion of *dGLYAT* blocks ectopic Egr or Hep-triggered JNK pathway activation. Furthermore, *dGLYAT* is required for physiological JNK activation-induced cell death, which is triggered by depletion of *puc* or *lgl*. Finally, loss of *dGLYAT* impedes activated JNK signaling-induced ROS production. Thus, these data not only represent the first *in vivo* function of dGLYAT in *Drosophila* development, but also suggest a role of GLYAT in regulating JNK signaling in mammals.

## Result and Discussion

### Loss of *dGLYAT* suppresses ectopic Egr-induced cell death in eye development

**Figure 1:**
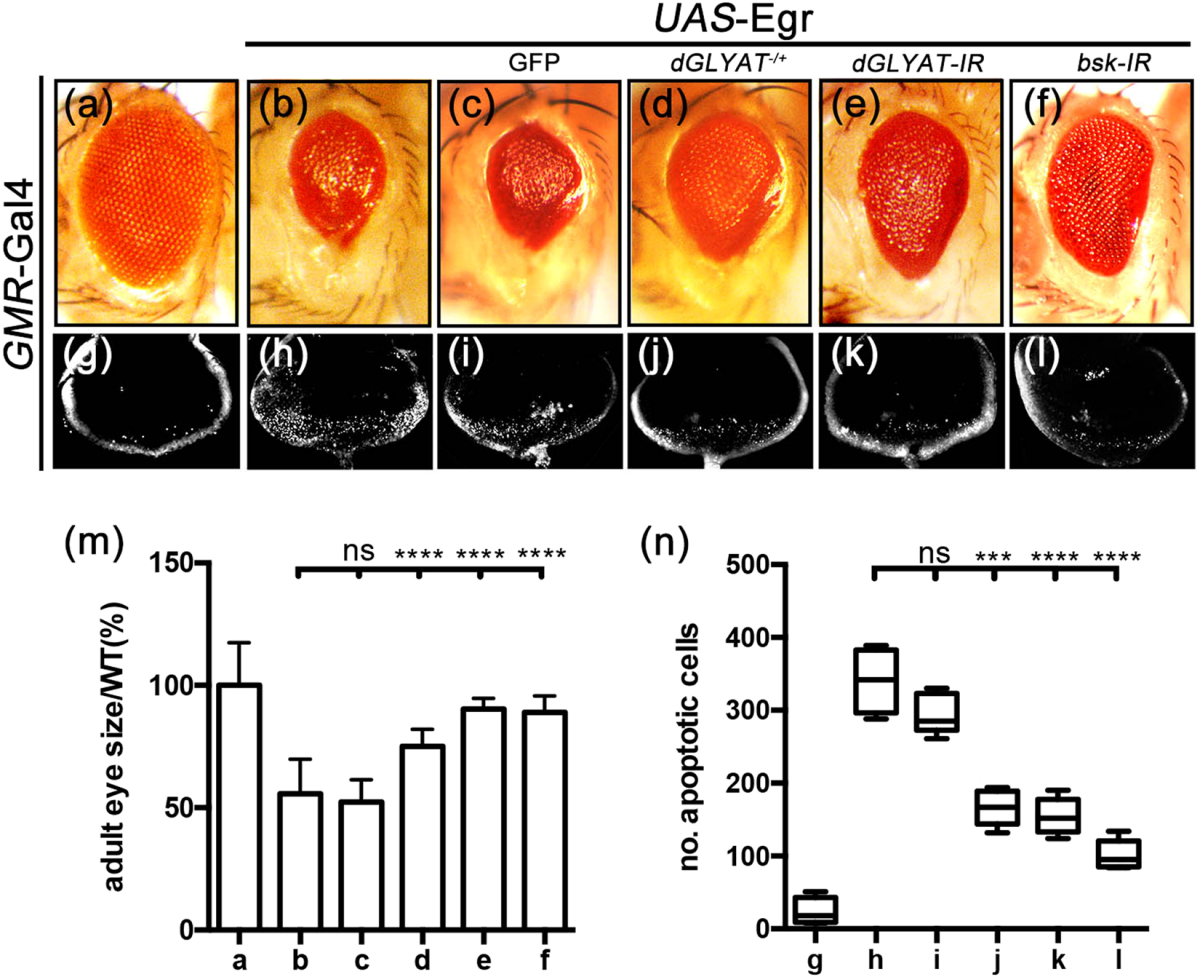
Loss of *dGLYAT* suppresses ectopic Egr-induced cell death in eye development. Light micrographs of *Drosophila* adult eyes (**a–f**) and fluorescent micrographs of third instar eyes discs (**g–l**) are shown. Compared with the *GMR*-Gal4 controls (**a,g**), *GMR* > Egr induces a small eye phenotype in adults (**b**) and massive cell death in 3^rd^ instar larval eye discs with AO staining (**h**). Both phenotypes are suppressed by mutation (**d,j**) or RNAi-mediated depletion (**e**,**k**) of *dGLYAT*, but remains unaffected by expressing GFP (**c**,**i**). Expression of *bsk-IR* serves as a positive control (**f**,**l**). (**m**) Statistics of eye sizes shown in (**a**–**e**) (a, n = 15; b, n = 11; c, n = 26; d, n = 20; e, n = 13; f, n = 14). (**n**) Statistics of AO-positive cell numbers shown in (**g**–**l**) (g, n = 5; h, n = 6; i, n = 12; j, n = 7; k, n = 7; l, n = 8). n.s., P > 0.05; ****P < 0.0001; ***P < 0.001.

Compared with the control (Fig. 1a), ectopic expression of TNF ortholog Egr in the developing eye driven by *GMR*-Gal4 (*GMR* > Egr) triggers JNK-dependent cell death and produces a small eye phenotype in adults (Fig. 1b)^22, 30–35^. We found the *GMR* > Egr eye phenotype was significantly suppressed by a mutation in *CG34010* (Fig. 1d), a novel gene whose function was previously unknown. *CG34010* encodes a *Drosophila* ortholog of glycine N-acyltransferase (GLYAT), and is referred to as *dGLYAT* hereafter. The mutant, *PBac{PB}CG34010* ^*c02982*^, has a piggyBac insertion into the second exon and generates a truncated protein that deletes most of the coding region including the critical Gcn5-related N-acetyltransferases (GNAT) domain. Thus, the mutant is most probably a null allele for *dGLYAT*. Interestingly, the mutant is homozygously viable, and does not produce any discernible phenotype, suggesting it is not essential for normal development. Furthermore, RNAi-mediated depletion of *dGLYAT* also suppressed the *GMR* > Egr induced small eye phenotype (Fig. 1e), compared with the expression of a *UAS*-GFP transgene that served as a negative control (Fig. 1c), confirming that *dGLYAT* is required for ectopic Egr-triggered morphological defect. Expression of RNAi-mediated depletion of *Bsk*, the *Drosophila* JNK ortholog, served as a positive control (Fig. 1f). Consistently, *GMR* > Egr-triggered cell death, indicated by acridine orange (AO) staining, posterior to the morphogenetic furrow (MF) in 3^rd^ instar eye discs (Fig. 1h), was significantly impeded by loss of *dGLYAT* or *Bsk* (Fig. 1j–l), but remained unaffected by the expression of GFP (Fig. 1i). The statistics of adult eye sizes (Fig. 1m) and apoptotic cell numbers in larval eye discs (Fig. 1n) were shown. Taken together, the above data suggest that *dGLYAT* is physiologically required for ectopic Egr-induced cell death in eye development.

### Loss of *dGLYAT* impedes ectopic Hep-induced cell death in eye development

**Figure 2:**
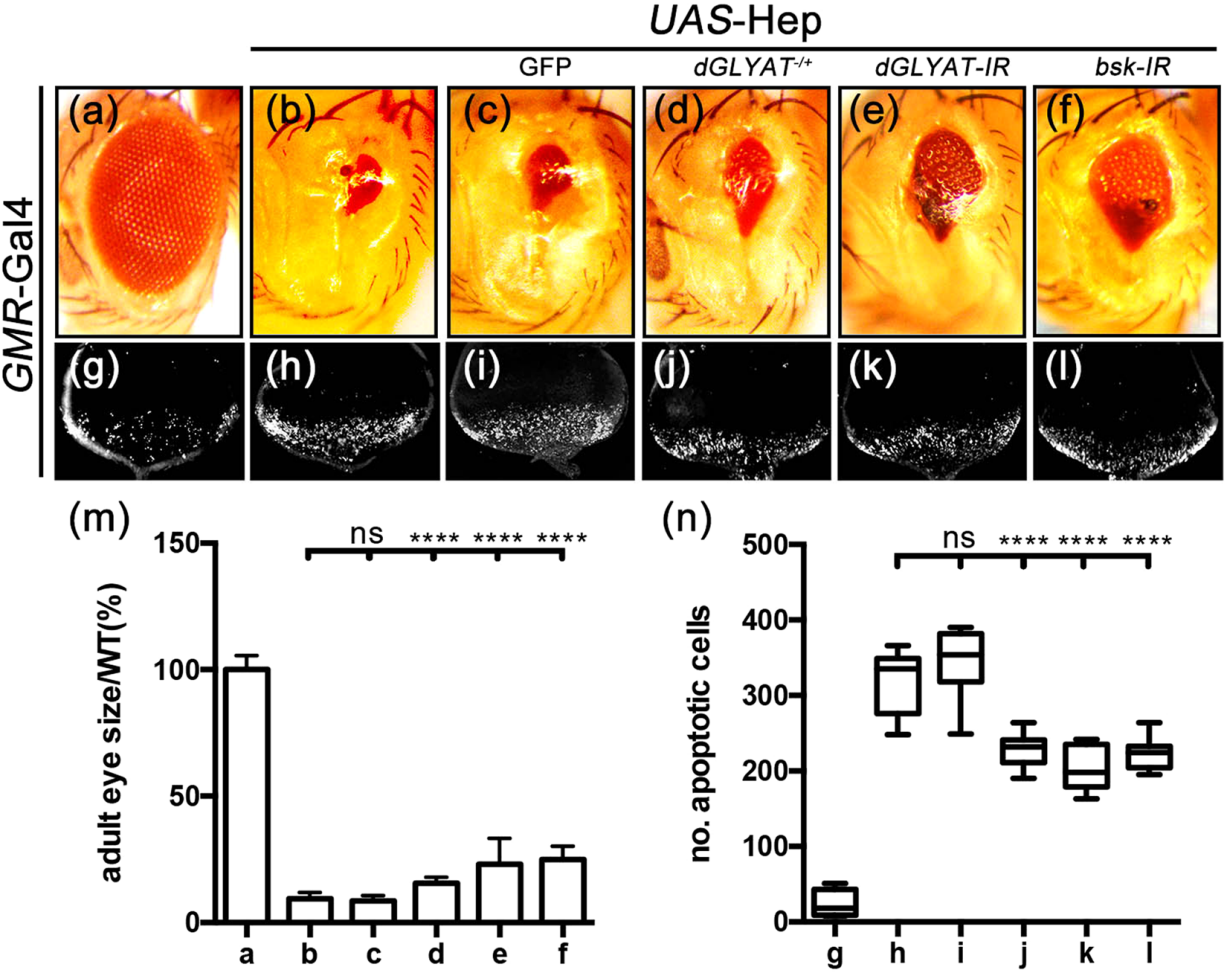
Loss of *dGLYAT* suppresses ectopic Hep-induced cell death in eye development. Light micrographs of *Drosophila* adult eyes (**a–f**) and fluorescent micrographs of third instar eyes discs (**g–l**) are shown. Compared with the *GMR*-Gal4 controls (**a**,**g**), *GMR* > Hep induces a small eye phenotype in adults (**b**) and extensive cell death in 3^rd^ instar larval eye discs (**h**). Both phenotypes are suppressed by mutation (**d**,**j**) or RNAi-mediated depletion (**e**,**k**) of *dGLYAT*, but remains unaffected by expressing GFP (**c**,**i**). Expression of *bsk-IR* serves as a positive control (**f**,**l**). (**m**) Statistics of eye sizes shown in (**a**–**e**) (a, n = 12; b, n = 51; c, n = 29; d, n = 16; e, n = 26; f, n = 20). (**n**) Statistics of AO-positive cell numbers shown in (**g**–**l**) (g, n = 5; h, n = 8; i, n = 8; j, n = 11; k, n = 8; l, n = 8). n.s., P > 0.05; ****P < 0.0001.

*GMR* > Egr triggers cell death via two independent pathways, the caspase pathway and the JNK pathway^34^. To examine whether *dGLYAT* is required for caspase-mediate cell death, we overexpressed *Drosophila* p53 (Dp53), a pro-apoptotic gene that triggers caspase-mediated cell death^36–39^, in the eye by *GMR*-Gal4. We found that *GMR* > Dp53-triggered small eye phenotype was not suppressed by loss of *dGLYAT* (Figure S1), suggesting *dGLYAT* is not involved in caspase-mediated cell death. To investigate the role of *dGLYAT* in JNK-mediated cell death, we expressed a constitutive active form of the *Drosophila* JNK kinase Hemipterous (Hep) in the developing eye. *GMR* > Hep^CA^ induces JNK-mediated cell death in eye discs (Fig. 2h) and produces a small eye phenotype in adults (Fig. 2b)^31, 33^. Both phenotypes were significantly suppressed by loss of *dGLYAT* or depletion of *Bsk*, but not the expression of GFP (Fig. 2c–f,i–n). Thus, *dGLYAT* is necessary for ectopic Hep-induced JNK-mediated cell death in eye development.

### Loss of *dGLYAT* inhibits JNK activation in eye discs

**Figure 3:**
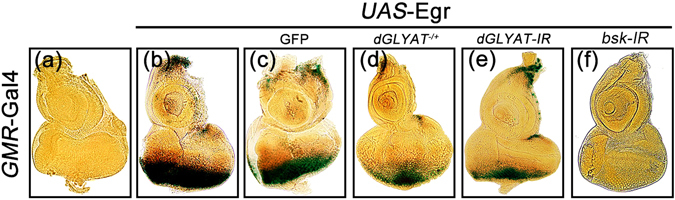
Loss of *dGLYAT* suppresses ectopic Egr-induced *puc* expression in eye discs. Light micrographs of *Drosophila* third instar eye discs with X-Gal staining are shown. Compared with the *GMR*-Gal4 control (**a**), ectopic Egr-induced *puc*-LacZ expression in eye discs (**b**) is suppressed by mutation (**d**) or *RNAi*-mediated depletion (**e**) of *dGLYAT*. Expression of GFP (**c**) and *Bsk-IR* (**f**) acts as a negative and a positive control (a, n = 4; b, n = 6; c, n = 8; d, n = 6; e, n = 5; f, n = 9).

The above data suggest that *dGLYAT* is necessary for JNK-mediated cell death in eye development, yet it remains unknown whether *dGLYAT* is required for JNK pathway activation. To address this, we checked the expression of *puc*-LacZ, a well-known readout of JNK signaling in *Drosophila* ^25, 40, 41^. We found that *GMR* > Egr induced strong *puc*-LacZ expression posterior to the morphogenetic furrow (MF) in the eye disc (Fig. 3b), which was remarkably inhibited by loss of *dGLYAT*. Again, expression of *Bsk* and GFP were served as a positive and negative controls, respectively (Fig. 3c–f). Hence, *dGLYAT* is necessary for JNK signaling activation in eye development.

### *dGLYAT* modulates JNK-mediated cell death in other tissues

**Figure 4:**
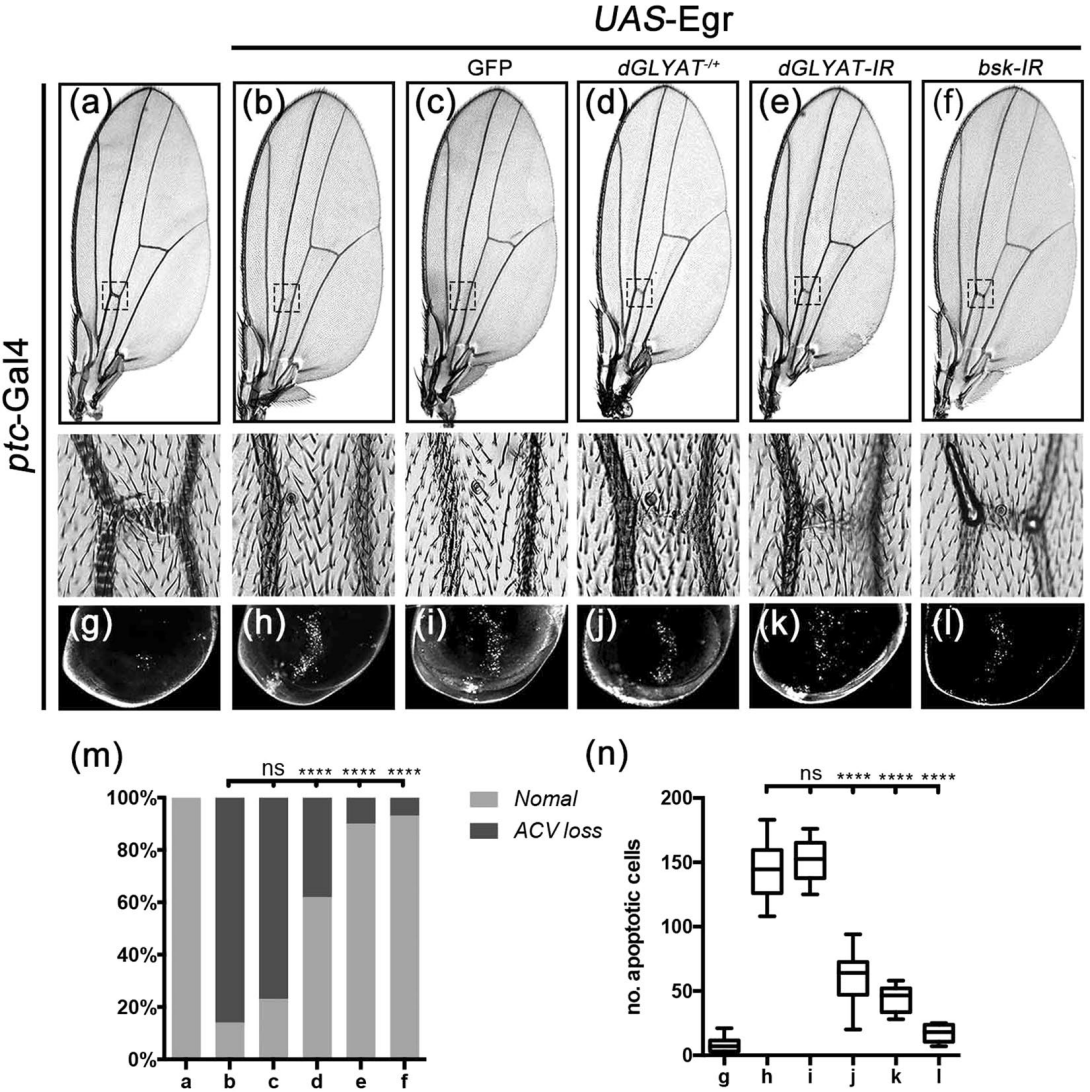
Loss of *dGLYAT* suppresses ectopic Egr-induced cell death in wing development. Light micrographs of *Drosophila* adult wings (**a–f**) and fluorescent micrographs of third instar wing discs (**g–l**) are shown. The lower panels are high magnification of boxed area in upper panels (**a**–**f**). Compared with the *ptc*-Gal4 controls (**a,g**), *ptc* > Egr induces a loss-of-ACV phenotype in adult wings (**b**) and extensive cell death along the A/P boundary in third instar larval wing discs (**h**), while remains unaffected by expressing GFP as a negative control (**c,i**). Both phenotypes are suppressed partially by mutation in *dGLYAT* (**d,j**) and strongly by RNAi-mediated knocking-down of *dGLYAT* (**e,k**). Expression of *Bsk-IR* acts as a positive control (**f,l**) (**m**) Statistics of the loss-of-ACV phenotype in (**a–f**) (a, n = 50; b, n = 56; c, n = 90; d, n = 88; e, n = 113; f, n = 80), (n) Statistics of the AO-positive cell number in (**g–l**) (g, n = 17; h, n = 12; i, n = 12; j, n = 21; k, n = 12; l, n = 10). n.s., P > 0.05; ****P < 0.0001.

To investigate whether *dGLYAT* modulates JNK-mediated cell death in other tissues, we examined the interaction between *dGLYAT* and JNK signaling in the developing wing, another important tissue frequently used for genetics studies. Ectopic expression of Egr driven by *ptc*-Gal4 (*ptc* > Egr) was able to induce extensive cell death along the anterior/posterior (A/P) compartment boundary in 3^rd^ instar larval wing discs (Fig. 4h) and produce loss of the anterior cross vein (ACV) phenotype in adult wings (Fig. 4b)^22, 42^. We found that both phenotypes were significantly suppressed by loss of *dGLYAT* or *Bsk*, but not the expression of GFP (Fig. 4c–f,i–n). Thus, *dGLYAT* is also required for ectopic Egr-triggered cell death in wing development.

**Figure 5:**
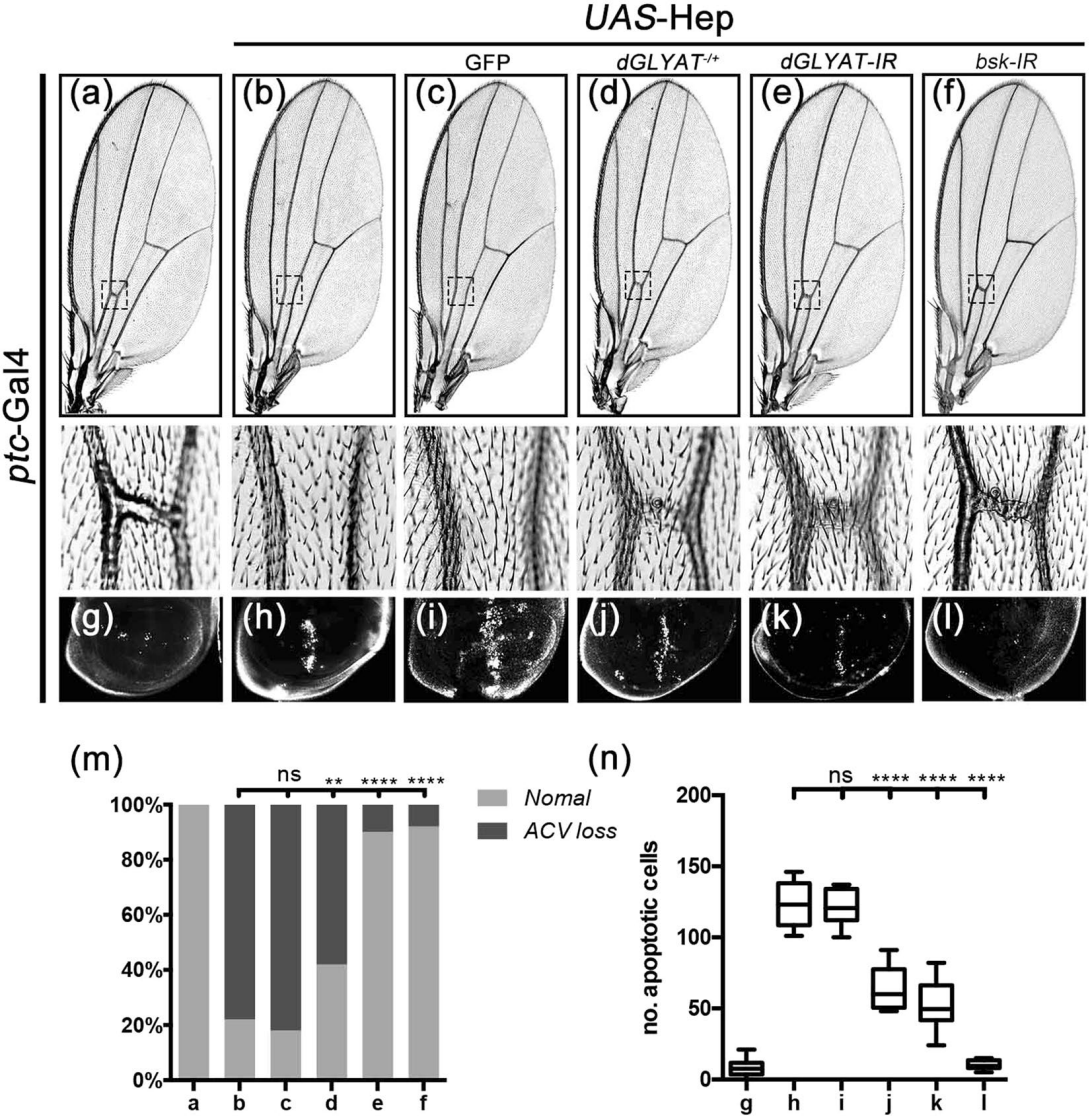
Loss of *dGLYAT* suppresses ectopic Hep-induced cell death in wing development. Light micrographs of *Drosophila* adult wings (**a–f**) and fluorescent micrographs of third instar wing discs (**g–l**) are shown. The lower panels are high magnification of boxed area in upper panels (**a–d**). Compared with the *ptc*-Gal4 controls (**a,g**), *ptc* > Hep induces a loss-of-ACV phenotype in adult wings (**b**) and extensive cell death along the A/P boundary in third instar larval wing discs (**h**), while remains unaffected by expressing GFP as a negative control (**c,i**). Both phenotypes are suppressed partially by mutation in *dGLYAT* (**d,j**) and strongly by RNAi-mediated knocking-down of *dGLYAT* (**e,k**). (**m**) Statistics of the loss-of-ACV phenotype in (**a–f**) (a, n = 70; b, n = 176; c, n = 110; d, n = 93; e, n = 146; f, n = 98). (**n**) Statistics of the AO-positive cell number in (**g–l**) (g, n = 16; h, n = 5; i, n = 7; j, n = 9; k, n = 10; l, n = 6). n.s., P > 0.05; ****P < 0.0001; **P < 0.01.

**Figure 6:**
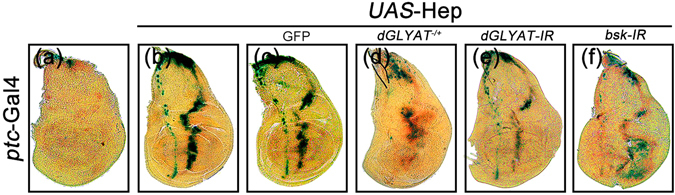
Loss of *dGLYAT* suppresses ectopic Hep-induced *puc* transcription in wing discs. Light micrographs of *Drosophila* third instar wing discs with X-Gal staining are shown. Compared with the *ptc*-Gal4 control (**a**), *ptc* > Hep-induced *puc*-LacZ expression in wing disc (**b**) is suppressed by mutation (**d**) or RNAi-mediated depletion of *dGLYAT* (**e**). Expression of GFP (**c**) and *Bsk-IR* (**f**) acts as a negative and a positive control (a, n = 8; b, n = 10; c, n = 5; d, n = 10; e, n = 11; f, n = 9).

Ectopic expression of Hep driven by *ptc*-Gal4 (*ptc* > Hep) induces JNK-mediated cell death in wing discs and generates a loss-of-ACV phenotype in adults^22, 32^, both of which were blocked by loss of *dGLYAT* (Fig. 5). Consistently, *ptc* > Hep-induced *puc*-LacZ activation along the A/P boundary in 3^rd^ instar larval wing discs (Fig. 6b)^23, 26, 30, 31^ was significantly inhibited by depleting *dGLYAT* or *Bsk*, but not the expression of GFP (Fig. 6c–f). Collectively, these data indicate that *dGLYAT* modulates JNK-mediated cell death in a non-tissue specific manner.

To investigate whether expression of *dGLYAT* is able to trigger JNK activation and cell death, we drove *dGLYAT* expression in the developing eye or wing by *GMR*-Gal4 or *ptc*-Gal4, respectively. We found that ectopic expression of *dGLYAT* did not trigger JNK signaling activation (Figure S2f) or cell death (Figures S2d and S2j) in the imaginal discs, and produced wild-type like eyes (Figure. S2b) and wings (Figure S2h) in the adult. Thus, expression of *dGLYAT* by itself is not sufficient to trigger JNK activation and cell death. Consistently, expression of *Bsk*, the fly JNK ortholog, or dTRAF2 that acts upstream of dTAK1, is not sufficient to induce JNK activation and cell death^23^. It remains to be explored whether expression of an activated form of *dGLYAT*, or co-expression of *dGLYAT* with its co-factor(s), is able to induce JNK activation and cell death in development.

### *dGLYAT* is required for physiological activation of JNK signaling

**Figure 7:**
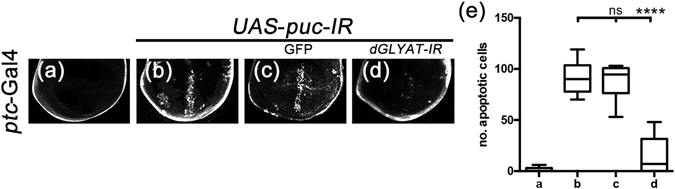
d*GLYAT* modulates loss of *puc*-induced cell death in wing discs. Light micrographs of *Drosophila* third instar wing discs (**a–d**) are shown. Compared with *ptc*-Gal4 controls (**a**), depletion of *puc* along the A/P boundary induces cell death in wing discs (**b**), which is suppressed by expressing a *dGLYAT-IR* (**d**), but remains unaffected by expressing GFP (c). (**e**) Statistics of the AO-positive cell numbers shown in (**a–d**) (a, n = 8; b, n = 8; c, n = 6; d, n = 9), n.s., P > 0.05; ****P < 0.0001.

The above data suggest that *dGLYAT* is important for ectopically activated JNK signaling-induced cell death, yet it remains unclear whether *dGLYAT* modulates the physiological functions of JNK signaling. To address this question, we knocked down *puc*, a negative regulator of JNK signaling, by the *ptc*-Gal4 driver. Depletion of *puc* induced robust cell death in third instar larval wing discs, as detected by AO staining (Fig. 7a and b). Intriguingly, the phenotype was significantly impeded by expressing a *dGLYAT RNAi*, but not GFP (Fig. 7c and d), suggesting *dGLYAT* is essential for physiologically activated JNK-induced cell death.

**Figure 8:**
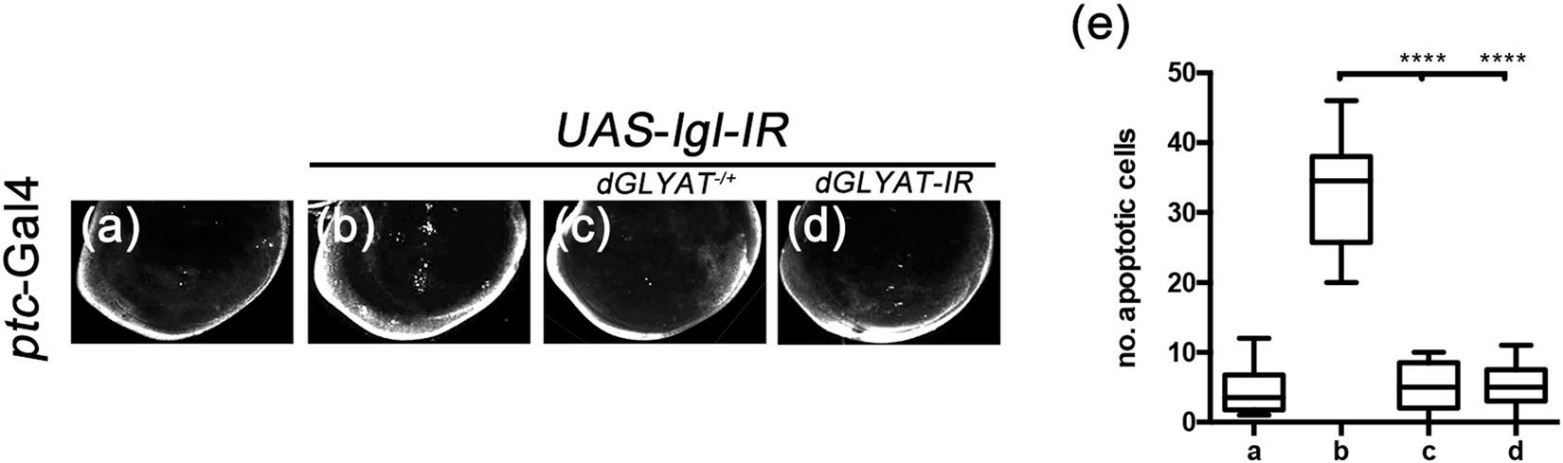
d*GLYAT* modulates loss of *lgl*-induced cell death in wing discs. Light micrographs of *Drosophila* wing discs are shown. Compared with the *ptc*-Gal4 control (**a**), depletion of *lgl* induces cell death along the A/P boundary (**b**), which is suppressed by mutation (**c**) or RNAi-mediated depletion (**d**) of *dGLYAT*. (**e**) Statistics of the AO positive cell numbers in (**a–e**) (a, n = 6; b, n = 10; c, n = 13; d, n = 7). ****P < 0.0001.

It has been reported that loss of cell polarity in wing disc epithelial results in JNK-mediated cell death^43, 44^. Consistently, knockdown the cell polarity gene *lethal giant larva* (*lgl*) by *ptc*-Gal4 promotes JNK-mediated cell death along the A/P boundary in third instar larval wing discs (Fig. 8b)^30, 31^. This phenotype was significantly suppressed in heterozygous *dGLYAT* mutant or by the expression of a *dGLYAT RNAi* (Fig. 8c–e). Therefore, *dGLYAT* modulates the physiological function of JNK signaling in development.

### *dGLYAT* regulates JNK-mediated ROS activation

**Figure 9:**
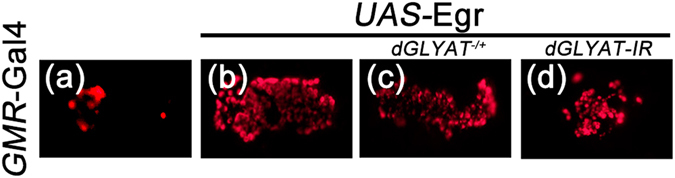
Loss of *dGLYAT* suppresses ectopic Egr-induced ROS activation in eye discs. Light micrographs of *Drosophila* eye discs are shown. Compared with the *GMR*-Gal4 control (**a**), *GMR* > Egr-induced ROS positive staining (**b**) is suppressed by mutation (**c**) or RNAi-mediated depletion (**d**) of *dGLYAT* (a, n = 8; b, n = 6; c, n = 5; d, n = 6).

Activated JNK signaling not only triggers cell death, but also stimulates Reactive Oxygen Species (ROS) in a feed-back loop^29^. To examine whether *dGLYAT* is crucial for JNK-mediated ROS activation, we detected ROS level in third instar larval eye discs by CellROX staining^29^. Consistent with previous study^28, 29, 45^, ectopic expression of Egr (*GMR* > Egr) promoted abundant ROS production (Fig. 9a and b), which was considerably suppressed by mutation or RNAi-mediated depletion (Fig. 9c and d) of *dGLYAT*, suggesting *dGLYAT* regulates JNK-mediated ROS activation *in vivo*.

Intriguingly, PFAMs, the catalytic products of hGLYATs, were reported to play a role in ROS activation^13, 14, 46^. However, the underlying mechanism has remained unknown, and a connection with JNK signaling has not been examined. Given the fact that both JNK pathway and its role in ROS have been highly conserved from *Drosophila* to human, it is plausible that GLYATs also regulate JNK signaling in mammals.

### Summary

dGLYAT contains a conserved GNAT domain and is supposed to function as an Acyl-CoA N-acyltransferase, yet its *in vivo* function has not been previously explored. In the present study, we identified dGLYAT as a crucial modulator of JNK pathway *in vivo* by using *Drosophila* as a model organism. We showed that loss of *dGLYAT* blocks not only ectopic Egr- or Hep-induced JNK activation and cell death, but also depletion-of-*puc* or *lgl*-triggered physiological JNK activation and cell death in development. In addition, loss of *dGLYAT* impedes JNK-dependent ROS activation. Thus, dGLYAT regulates multiple physiological functions of JNK signaling *in vivo*, yet the molecular mechanism by which dGLYAT regulates JNK pathway remains unknown, and should be addressed by further investigations.

## Materials and Methods

### *Drosophila* Genetics and Stocks

All stocks were raised on a standard cornmeal and agar medium at 25 °C unless otherwise indicated. For experiments involving *tub*-Gal80^ts^, eggs were allowed to develop at 25 °C for 2–3 days, then transferred to 29 °C for 2 days to inactivate Gal80.

*ptc*-Gal4^42^, *GMR*-Gal4^47^, *UAS*-Egr^Regg119^, *UAS*-GFP and *UAS*-Hep^CA^ ^48^, *UAS*-Dp53^33^, *tub*-Gal80^ts^ and *puc* ^*E69*33^ have been used previously; *UAS-puc-IR* (V3018) was obtained from Vienna Drosophila RNAi Center; *UAS-bsk-IR* (NIG5680R-2) was obtained from National Institute of Genetics (NIG-FLY); *PBac{PB}CG34010* ^*c02982*^ was obtained from Harvard (the Exelixis Collection); *UAS-dGLYAT-IR* was obtained from Tsinghua Fly Center.

### Acridine orange staining

Eye and wing discs were dissected from 3^rd^ instar larvae in 1 × PBS (phosphate-buffered saline) and incubated in 1 × 10^−5^ M acridine orange (AO) for 5 minutes at room temperature^34^.

### X-gal staining

Wing and eye discs were dissected from third instar larvae in PBST (1 × PBS pH 7.0, 0.1% Triton X-100) and stained for β-galactosidase activity as described^49^.

### Microscopy and phenotype analysis

Flies of indicated genotypes were collected and frozen in −80 °C. Wings were dissected and mounted on the slide in the alcohol/glycerol (1:1) medium, and flies were mounted in the alcohol on 3% agarose plate. Image of wings were captured with Olympus microscope BX51, and light image of eyes were captured with Olympus stereo microscope SZX16^32^.

### Statistical analysis

Results are presented in bar graphs and box graphs created with GraphPad Prism 6. For loss-of-ACV phenotype, statistics were analyzed by chi-square test. For AO staining and area of eye size, one-way ANOVA with Bonferroni’s multiple comparison tests are used to calculate statistical significance. P-values are included in the relevant figure legends.

### ROS staining

The level of ROS was detected by CellROX (Life Technologies). Eye discs were dissected from third instar larvae in *Drosophila* cell media, incubated in 5 μM CellRox for 15 minutes, rinsed in PBS, fixed in 3.7% formaldehyde for 5 minutes, and mounted in 80% glycerol for imaging^45^.

## Electronic supplementary material

Supplementary Information
