# Supplementary material for: GLYAT regulates JNK-mediated cell death in Drosophila
Source: Sci Rep. 2017 Jul 12;7:5183. doi: 10.1038/s41598-017-05482-y (PMC5507861; doi:10.1038/s41598-017-05482-y)
Supplement: Supplementary file 1 — Supplementary Information [file 41598_2017_5482_MOESM1_ESM.pdf]

GLYAT regulates JNK-mediated cell death in *Drosophila*

Pu Ren[1], Wenzhe Li[1] and Lei Xue[1,*]

Supplementary information

Ren et al., Fig. S1

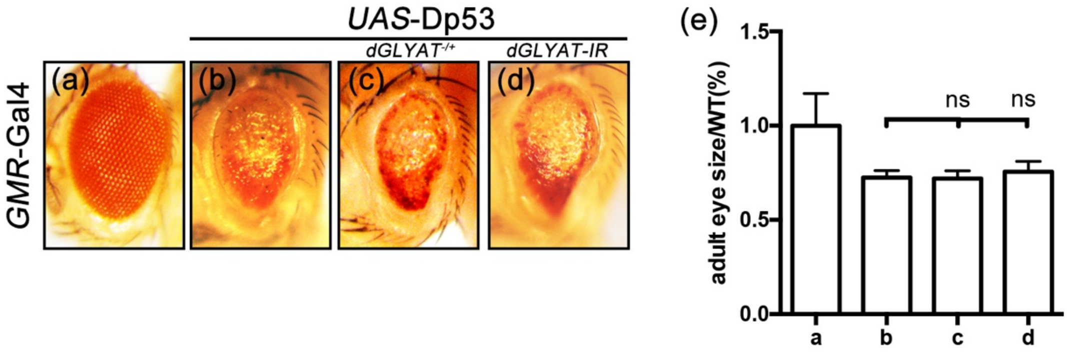

Figure S1 Loss of *dGLYAT* does not suppress caspase-mediated cell death.

Light micrographs of *Drosophila* adult eyes are shown. Compared with the *GMR*-Gal4 control (a), *GMR*>Dp53 induces a small eye phenotype (b), which is not suppressed by mutation in *dGLYAT* (c) or expression of a *dGLYAT-IR* (d). (e) Statistics of the eye sizes shown in a-d (a, n=14; b, n=20; c, n=22; d, n=22). n.s., P>0.05.

Ren et al., Fig. S2

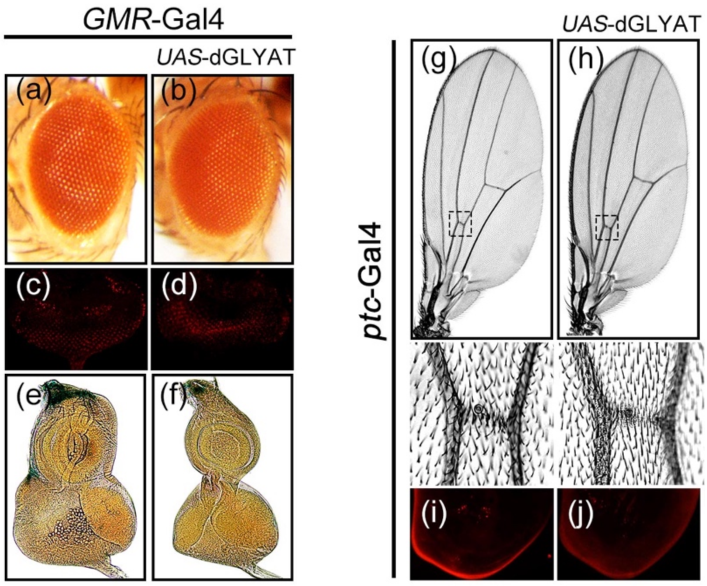

Figure S2 Expression of *dGLYAT* does not induce cell death and JNK activation.

Light micrographs of *Drosophila* adult eyes (a, b) and wings (g, h), and fluorescent micrographs of third instar eye (c, d) and wing (i, j) discs are shown. Compared with the *GMR*-Gal4 (a, c) or *ptc*-Gal4 (g, i) controls, expression of *dGLYAT* does not induces a small eye (b) or loss-of-ACV (h) phenotype in adults, or massive cell death in 3rd instar larval eye (d) or wing (j) discs. Compared with the *GMR*-Gal4 control (e), expression of *dGLYAT* does not induce *puc*-LacZ expression in eye discs (f).

<u>Detailed Genotypes</u>

Figure 1

(a, g) *GMR*-Gal4/+

(b, h) *UAS*-Egr$^{\mathrm{Regg1}}$/+; *GMR*-Gal4/+

(c, i) *UAS*-Egr$^{\mathrm{Regg1}}$/*UAS*-GFP; *GMR*-Gal4/+

(d, j) *UAS*-Egr$^{\mathrm{Regg1}}$/*dGLYAT*^{C02982}; *GMR*-Gal4/+

(e, k) *UAS*-Egr$^{\mathrm{Regg1}}$/+; *GMR*-Gal4/ *UAS-dGLYAT-IR*

(f, l) *UAS*-Egr$^{\mathrm{Regg1}}$/+; *GMR*-Gal4/ *UAS-bsk-IR*

Figure 2

(a, g) *GMR*-Gal4/+

(b, h) *GMR*-Gal4 *UAS*-Hep^{CA} *UAS-bsk-IR*/+

(c, i) *UAS*-GFP/+; *GMR*-Gal4 *UAS*-Hep^{CA} *UAS-bsk-IR*/+

(d, j) *dGLYAT*^{C02982}/+; *GMR*-Gal4 *UAS*-Hep^{CA} *UAS-bsk-IR*/+

(e, k) *GMR*-Gal4 *UAS*-Hep^{CA} *UAS-bsk-IR*/ *UAS-dGLYAT-IR*

(f, l) *GMR*-Gal4 *UAS*-Hep^{CA} *UAS-bsk-IR*/ *UAS-bsk-IR*

Figure 3

(a) *GMR*-Gal4/+

(b) *GMR*-Gal4 *UAS*-Egr/+; *puc*^{E69}/+

(c) *GMR*-Gal4 *UAS*-Egr/ *UAS*-GFP; *puc*^{E69}/+

(d) *GMR*-Gal4 *UAS*-Egr/ *dGLYAT*^{C02982}; *puc*^{E69}/+

(e) *GMR*-Gal4 *UAS*-Egr/+; *puc*^{E69}/ *UAS-dGLYAT-IR*

(f) *GMR*-Gal4 *UAS*-Egr/+; *puc*^{E69}/ *UAS-bsk-IR*

Figure 4

(a, g) *ptc*-Gal4/+

(b, h) *ptc*-Gal4 *UAS*-Egr/+; *tub*-Gal80^{ts}/+

(c, i) *ptc*-Gal4 *UAS*-Egr/ *UAS*-GFP; *tub*-Gal80^{ts}/+

(d, j) *ptc*-Gal4 *UAS*-Egr/ *dGLYAT*^{C02982}; *tub*-Gal80^{ts}/+

(e, k) *ptc*-Gal4 *UAS*-Egr/+; *tub*-Gal80^{ts}/ *UAS-dGLYAT-IR*

(f, l) *ptc*-Gal4 *UAS*-Egr/+; *tub*-Gal80^{ts}/ *UAS-bsk-IR*

Figure 5

(a, g) *ptc*-Gal4/+

(b, h) *ptc*-Gal4 *UAS*-Hep/+; *puc*^{E69}/+

(c, i) *ptc*-Gal4 *UAS*-Hep/ *UAS*-GFP; *puc*^{E69}/+

(d, j) *ptc*-Gal4 *UAS*-Hep/ *dGLYAT*^{C02982}; *puc*^{E69}/+

(e, k) *ptc*-Gal4 *UAS*-Hep/+; *puc*^{E69}/ *UAS-dGLYAT-IR*

(f, l) *ptc*-Gal4 *UAS*-Hep/+; *puc*^{E69}/ *UAS-bsk-IR*

Figure 6

(a) *ptc*-Gal4/+

(b) *ptc*-Gal4 *UAS*-Hep/+; *puc*^{E69}/+

(c) *ptc*-Gal4 *UAS*-Hep/ *UAS*-GFP; *puc*^{E69}/+

(d) *ptc*-Gal4 *UAS*-Hep/ *dGLYAT*^{C02982}; *puc*^{E69}/+

(e) *ptc*-Gal4 *UAS*-Hep/+; *puc*^{E69}/ *UAS-dGLYAT-IR*

(f) *ptc*-Gal4 *UAS*-Hep/+; *puc*^{E69}/ *UAS-bsk-IR*

Figure 7

(a) *ptc*-Gal4/+

(b) *ptc*-Gal4 *UAS-puc-IR*/+

(c) *ptc*-Gal4 *UAS-puc-IR*/ *UAS*-GFP

(d) *ptc*-Gal4 *UAS-puc-IR*/+; *UAS-dGLYAT-IR*/+

Figure 8

(a) *ptc*-Gal4/+

(b) *ptc*-Gal4/+; *UAS-lgl-IR*/+

(c) *ptc*-Gal4/ *dGLYAT*^{C02982}; *UAS-lgl-IR*/+

(d) *ptc*-Gal4/+; *UAS-lgl-IR*/ *UAS-dGLYAT-IR*

Figure 9

(a) *GMR*-Gal4/+

(b) *UAS*-Egr^{Regg1}/+; *GMR*-Gal4/+

(c) *UAS*-Egr^{Regg1}/ *dGLYAT*^{C02982}; *GMR*-Gal4/+

(d) *UAS*-Egr^{Regg1}/+; *GMR*-Gal4/ *UAS-dGLYAT-IR*

Figure S1

(a) *GMR*-Gal4/+

(b) *GMR*-Gal4 *UAS*-Dp53/+

(c) *GMR*-Gal4 *UAS*-Dp53/ *dGLYAT*^{C02982}

(d) *GMR*-Gal4 *UAS*-Dp53/+; *UAS-dGLYAT-IR*/+

Figure S2

(a, c) *GMR*-Gal4/+

(b, d) *GMR*-Gal4/ *UAS*-dGLYAT

(e) *GMR*-Gal4/+

(f) *GMR*-Gal4/ *UAS*-dGLYAT; *puc*^{E69}/+

(g, i) *ptc*-Gal4/+

(h, j) *ptc*-Gal4/ *UAS*-dGLYAT
